# Supplementary figures and images for: Expression of LTR and LINE1 transposable elements defines atypical teratoid/rhabdoid tumor subtypes
Source: Acta Neuropathol Commun. 2025 Jul 22;13:159. doi: 10.1186/s40478-025-02078-w (PMC12285028; doi:10.1186/s40478-025-02078-w)

Suppl. Figure 1

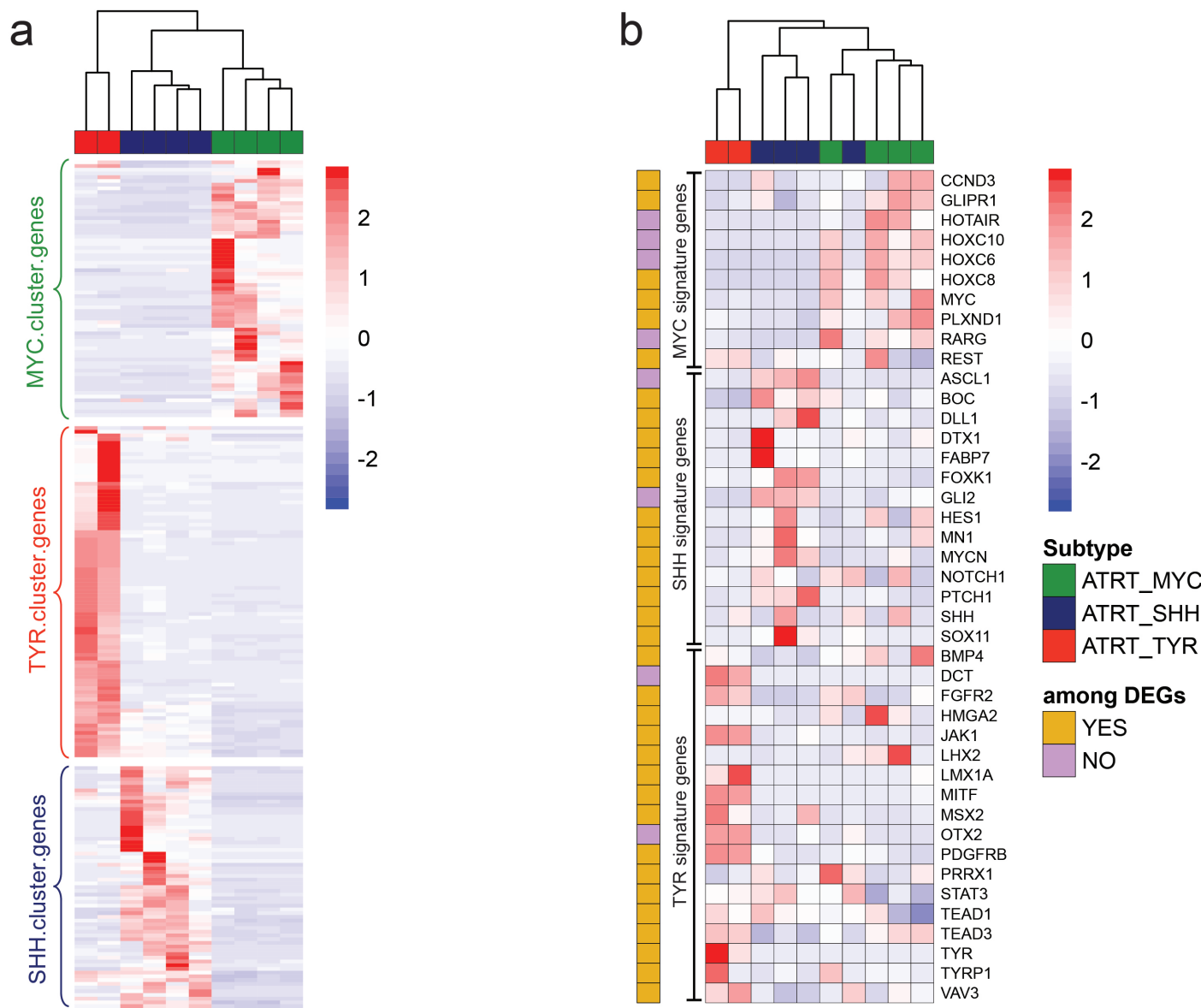

Suppl. Figure 2

a

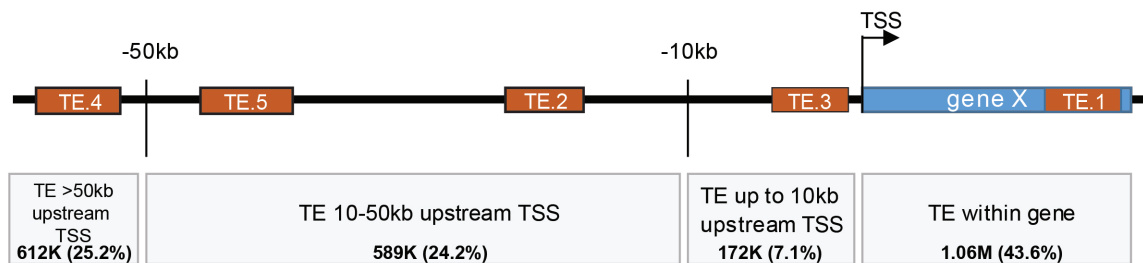

b

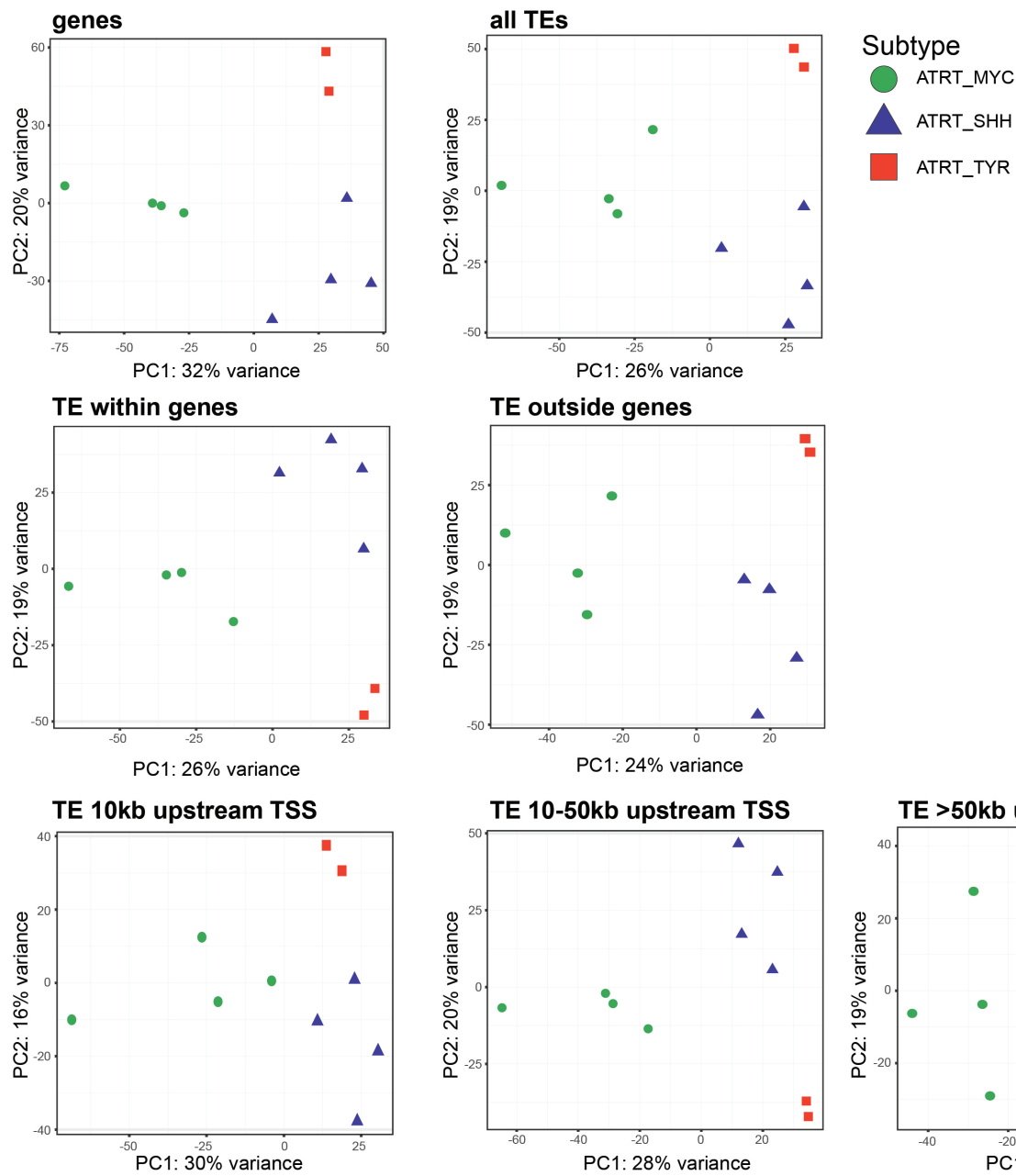

Suppl. Figure 3

# LINE1

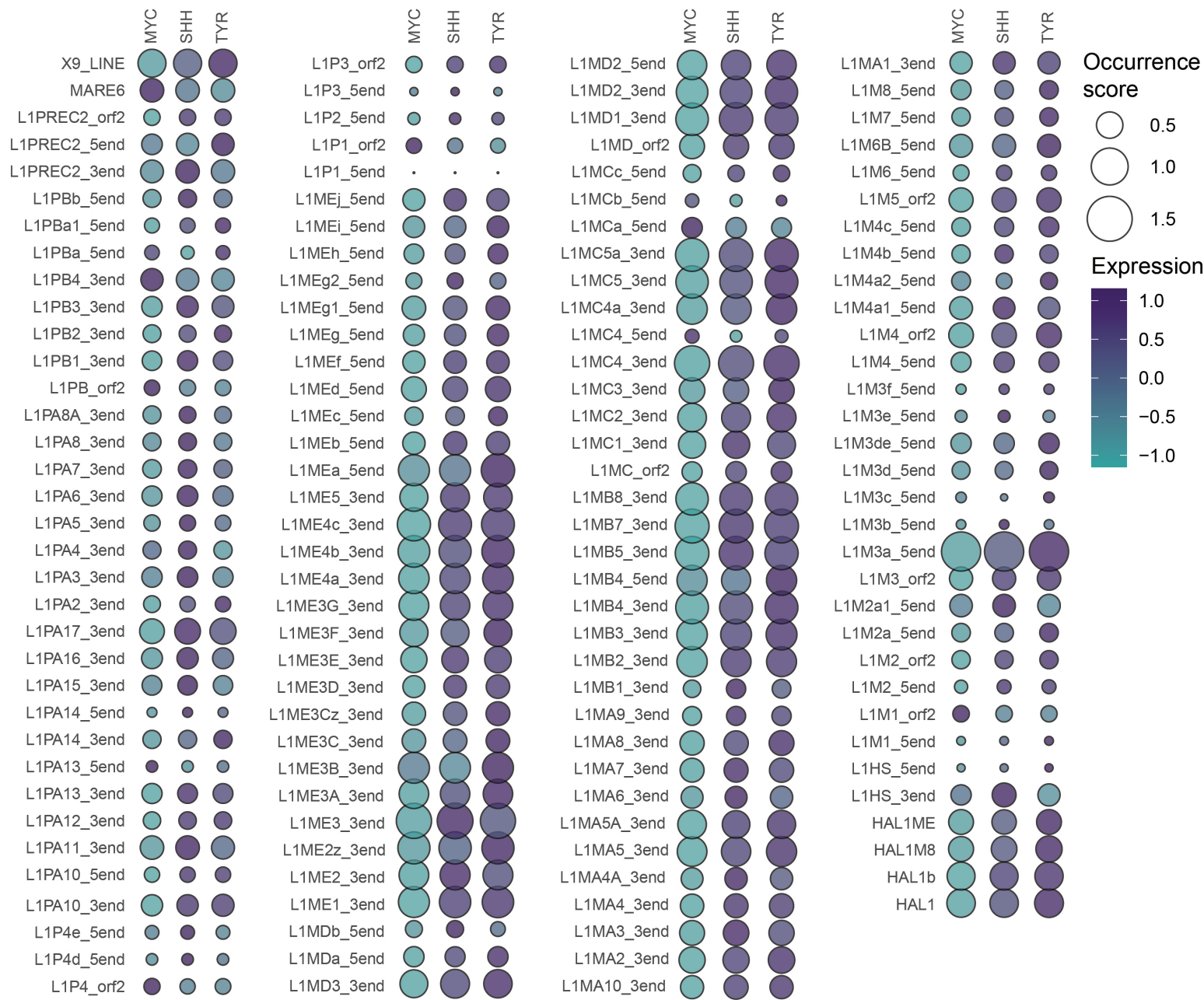

continues on next column

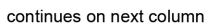

Supplement: Supplementary file 1 — Additional file1 (PDF 23591 kb) [file 40478_2025_2078_MOESM1_ESM.pdf]
