# Supplementary material for: Expression of LTR and LINE1 transposable elements defines atypical teratoid/rhabdoid tumor subtypes
Source: Acta Neuropathol Commun. 2025 Jul 22;13:159. doi: 10.1186/s40478-025-02078-w (PMC12285028; doi:10.1186/s40478-025-02078-w)
Supplement: Supplementary file 2 — Additional file2 (DOCX 12 kb) [file 40478_2025_2078_MOESM2_ESM.docx]

**Suppl. Fig. 1: Signature gene expression analysis in primary ATRT samples**

Pairwise differential gene expression (DEG) analysis between all three ATRT subtypes (MYC, SHH and TYR). Cutoffs for DEGs set to log2FC >|2|, padj < 1x10E-06. a) Unsupervised hierarchical clustering of all differentially expressed genes combined. b) Unsupervised hierarchical clustering of 42 ATRT signature genes being de-regulated in specific ATRT subtypes.

**Suppl. Fig. 2: Sample clustering based on gene and TE transcription profile subsets**

a) Representation of TE bins with respect to gene transcriptional start sites. b) Principal component analysis of ATRT samples based on gene and TE transcription, respectively. PCA based on vst-transformed counts, plotted 500 most variable genes/TEs.

**Suppl. Fig. 3: Differences in LINE1 transcription at subfamily level**

Relative normalized TE counts were summarized at TE subfamily level (expression score). Occurrence score represents the number of expressed loci per subfamily [in %].

**Suppl. Fig. 4: Differences in LTR transcription at subfamily level**

Relative normalized TE counts were summarized at TE subfamily level (expression score). Occurrence score represents the number of expressed loci per subfamily [in %].

**Suppl. Fig. 5: Differential TE transcription analysis**

Relative fractions of clusterTEs at LTR family a) and subfamily level b), as well as fractions of clusterTEs for LINE1 subfamilies c). Due to the large number of subfamilies, LTR and LINE1 subfamilies were grouped based on their annotation name as indicated in the legend.
